# The Effect of the Insurance Amendment Act on Hospital Finance

**Published:** 1913-11-01

**Authors:** 


### OUR NATIONAL INSURANCE ACT DEPARTMENT.

### LXXII.—The Effect of the Amendment Act on Hospital Finance.

It is becoming increasingly evident that the approved societies stand to gain by a better understanding with the medical profession, including panel and non-panel practitioners. As an instance it may be cited that the appointment, little more than a month ago, by the London Insurance Committee of medical referees, whose services should be at the disposal of the approved societies having members within the Committee area, has proved so successful that it is intended to extend the system. The medical referees have reported on cases submitted to them for special examination, and in a considerable proportion they have certified the members as fit for work, while a substantial proportion of those notified to submit themselves for examination have declined to do so, and have declared off the funds of their societies on their own initiative. Every case of this description speaks to the absolute necessity of complete harmony between the doctors and the societies. The approved societies have been formed for the mutual insurance of their members against sickness, and when a member is ill, and entitled to sickness benefit, the society is there to satisfy the claim. But the societies have a right to demand from the medical profession that such persons who are fit for work, and therefore not entitled to benefit, should not, through indulgence or laxity, be allowed to remain on the funds. On the other hand the doctors have a right to expect generous treatment from the societies, both directly and through the Insurance Committees, and to a recognition of the difficult situations in which they are frequently placed in determining whether to allow a claim to proceed or not. In course of time it is to be hoped that a complete understanding will be arrived at between the parties, to the mutual advantage of all concerned.

### Financial Recognition of Hospital Treatment.

One very obvious way in which that mutual understanding could be strengthened is by the fuller recognition on the part of the approved societies of the work done by the voluntary hospitals for the good of their members. It may be said at the present time that, with minor exceptions, the good work of the hospitals is absolutely disregarded by the approved societies in the important matter of contributing to the cost of treatment of members in hospital. The societies must clearly recognise, and we think they appreciate, the unspeakable advantages that accrue through the existence of the voluntary hospitals, but it is shortsighted policy for them to allow even the possibility that the hospitals may have to close their wards to insured persons through lack of necessary funds. And yet this is virtually what is happening. With access to their panel doctors more persons than ever are already being sent to the hospitals for treatment in the early stages of serious illness, while, on the other hand, the agencies for raising funds for the hospitals are reporting diminished collections from those in former days, with every risk of still further reduced voluntary assistance in future. Clearly the matter cannot be left indefinitely in this position. The hospitals have, it is urged, the right to look to the approved societies for substantial and adequate financial support in consideration of the benefits conferred upon sick members, and thus to the societies as a whole.

It has to be admitted, however, that the matter is not one which admits of easy solution. The approved societies are not yet, and will not for some time be, in a position to know exactly how they stand in regard to the sufficiency of their funds to meet their liabilities, and it is, perhaps, not to be wondered at that, in the circumstances, they should be chary of incurring financial responsibilities other than those they are bound to recognise under the compulsory provisions of the Acts. It is, therefore, not entirely the fault of the societies that they appear to disregard the claims of the hospitals. The blame is rather on the Government for their attitude towards the question when it was before Parliament.

### Futility of Permission to Make Agreements.

The unsatisfactory character of the provisions of Section 12 of the Act of 1911 has more than once been mentioned in our columns. No clearer statement of the futility of the permission accorded to the approved societies of entering into agreements with the hospitals has been made than in the Report of the London Hospital, extracts from which were published in our issue of June 14 last (p. 332). It was hoped that the Amendment Act would provide an opportunity for rectifying the matter, but although the repeal of the section was moved, the proposal was defeated. The account of the discussion in Committee over this section, as set forth in The Hospital for August 2 (p. 525), is worth noting, as it clearly indicates the attitude of the Government to the whole question of the hospital treatment of insured persons. Instead of the repeal of this ineffective section the Committee accepted the amendment proposed by Mr. Masterman, without notice, and the effect of that amendment, as it now appears in Section 15 of the Act of 1913, is to make it practically impossible for the approved societies to contribute to hospital funds.

### The Insured Person with no Dependants.

When an insured person, who has dependants, receives treatment in hospital no question arises under either Act, for his society is under obligation to pay the sickness benefit to such dependants. When, however, there were no dependants, and there was no agreement between the approved society and the hospital, Section 12 of the Act of 1911 provided that the sickness benefit to which the member would have been entitled, but for the fact that he was receiving treatment in hospital, was not to be paid to him during the time that he was an inmate, though it might have been used in providing surgical appliances or otherwise for his benefit. The Amendment Act has now come along and provides (Section 15) that in similar circumstances the sickness benefit shall be paid to the member in cash on leaving the institution in a lump sum, or by instalments, as the society or Insurance Committee (in the case of deposit contributors) thinks fit. Formerly there were doubts as to the legal rights of the societies to make such payments, but these have now been set at rest, and as from October 13, when the particular section came into operation, the societies will pay in cash, probably in a lump sum in most cases, any balance of the sickness benefit which has not been expended during the time the insured person has been in hospital. Seeing, then, that a society is now compelled to pay to the member on leaving hospital, in default of agreement with the hospital to the contrary, there is less hope than formerly of being able to obtain a contribution from the funds of the society as such. The hospitals will know, however, that the patient will be receiving a sum of money on leaving the institution, and while not making an actual charge, it could reasonably ask for a substantial proportion of that sum as a measure of compensation for the treatment given. Whatever the contribution of the particular insured person may be, it will be entirely voluntary on his part, and for this reason little reliance can be placed on such contributions as regular income.

### An Awkward Phrase.

One further point, and that a most important one, arises out of the precise wording adopted in Section 15 of the Amendment Act. The words employed are " . . . if and so far as it [the benefit] is not paid or applied in accordance with the provisions of that section [Section 12, Act 1911] while the person . . . is an inmate of any . . . hospital . . . may, if the society . . . administering the benefit thinks fit, be applied in the provision of any surgical appliances required for the person or otherwise for his benefit *after he ceases to be an inmate*, or, if it is not so expended, shall be paid in cash to the person on leaving the institution, in a lump sum or in instalments as the society or committee thinks fit." The point to be noted here is that while under the principal Act there was no restriction as to the application of the money for the benefit of the insured person, except from the payment in hard cash, there is now the qualification which we have italicised. It is somewhat doubtful whether this may not even limit the application of the money to the provision of surgical appliances required *after he ceases to be an inmate.* If this be so the hospitals will be distinctly worse off than formerly. In any case the words " otherwise for his benefit " are undoubtedly qualified by the phrase in question, so that it is clear, for instance, that any articles of clothing required by the patient while in hospital could not as formerly be provided for him out of his sickness benefit.

All this tends to make confusion worse confounded, but it strengthens the case of those who claim that the matter should not be left to the chance of arriving at a satisfactory voluntary arrangement with the individual insured person.

If the approved societies could be induced to enter into agreements with the hospitals for the treatment of their members the whole difficulty would disappear. Draft forms of agreement, suitable to the occasion, have been prepared by the Insurance Commissioners, as we pointed out so long ago as June 21 last (p. 366).

### Is it now Possible to Obtain Agreements with Approved Societies?

This brings us back to our introductory remarks. The approved societies have not been slow to appreciate the work of the medical referees appointed by the London and other Insurance Committees. Why, then, should there be any further difficulty in making satisfactory arrangements with the hospitals for the benefit of members? The present time is propitious for re-opening the question, in view of the fact that Section 15 of the Amendment Act has only just come into operation. All the larger approved societies are daily becoming more and more concerted in their action, and representations, carefully and tactfully made by hospital authorities in the proper quarters, should result in the vast majority of such societies effecting agreements with the hospitals for the treatment of members. Conditions have changed since the London Hospital approached the societies in the early part of the year, and we feel confident that there is now more chance of success.

NOTE.—Questions and Correspondence on all doubtful points are invited, and should be addressed to the Insurance Editor, The Hospital, 28 Southampton Street, Strand.

